# *Vibrio vulnificus* VvhA induces autophagy-related cell death through the lipid raft-dependent c-Src/NOX signaling pathway

**DOI:** 10.1038/srep27080

**Published:** 2016-06-02

**Authors:** Eun Ju Song, Sei-Jung Lee, Hyeon Su Lim, Jun Sung Kim, Kyung Ku Jang, Sang Ho Choi, Ho Jae Han

**Affiliations:** 1 Department of Veterinary Physiology, College of Veterinary Medicine, Research Institute for Veterinary Science, and BK21 PLUS Creative Veterinary Research Center, Seoul National University, Seoul 08826, Korea; 2 National Research Laboratory of Molecular Microbiology and Toxicology, Department of Agricultural Biotechnology, and Center for Food Safety and Toxicology, Seoul National University, Seoul 08826, Korea

## Abstract

VvhA, a virulent factor of *Vibrio (V.*) *vulnificus*, induces acute cell death in a destructive manner. Autophagy plays an important role in cell death, but the functional role of VvhA in autophagy-related cell death has not been elucidated yet. We found that rVvhA significantly increased LC3 puncta formation and autophagic flux in promoting the cell death of human intestinal epithelial Caco-2 cells. The cell death induced by rVvhA was independent of lysosomal permeabilizaton and caspase activation. rVvhA induced rapid phosphorylation of c-Src in the membrane lipid raft, which resulted in an increased interaction between lipid raft molecule caveolin-1 and NADPH oxidase (NOX) complex Rac1 for ROS production. NOX-mediated ROS signaling induced by rVvhA increased the phosphorylation of extracellular signal-regulated kinase (ERK) and eukaryotic translation initiation factor 2α (eIF2α) which are required for mRNA expression of Atg5 and Atg16L1 involved in autophagosome formation. In an *in vivo* model, VvhA increased autophagy activation and paracellular permeabilization in intestinal epithelium. Collectively, the results here show that VvhA plays a pivotal role in the pathogenesis and dissemination of *V. vulnificus* by autophagy upregulation, through the lipid raft-mediated c-Src/NOX signaling pathway and ERK/eIF2α activation.

*Vibrio (V.*) *vulnificus,* an etiologic agent of seafood-associated fatalities worldwide, has the ability to cause lethal infections including primary septicemia, wound infection and gastroenteritis^1^,^2^,^3^. VvhA, an extracellular haemolysin pore-forming toxin produced by *V. vulnificus*, is actively produced *in vivo*, and plays an essential role in human infection. During intestinal infection, in addition to a hemolytic activity, the cytotoxic effects of VvhA contribute to the invasion of the bacterium into the blood stream^4^,^5^. However, it was previously shown that the virulent effects of VvhA were not evident in mice infected with a *vvhA* mutant^6^. Due to the controversy about the *in vivo* and *in vitro* roles of VvhA in regulating the virulent effects of *V. vulnificus*, a previous report has suggested that VvhA may not be responsible for the lethality but may play a role in the survival of *V. vulnificus*^7^. Many pathogens can survive in host cells by modulating the host defense mechanism like the autophagy system^8^. However, the molecular mechanism behind the interaction between VvhA and the defense system for host cell death during *V. vulnificus* infection has not been clarified yet.

Autophagy is a conserved physiological pathway consisting of several steps including initiation, autophagosome formation, fusion with lysosome and degradation^9^,^10^. Although autophagy maintains cellular homeostasis and protects the host cell from harmful stimuli, autophagy flux is linked to unpleasant consequences such as cell death in certain conditions of cellular stress^11^,^12^. Autophagic cell death is a type II programmed cell death which is referred to as the impairment of the autophagy pathway. Specifically, autophagic cell death occurs when the cell is devastated with infection or when apoptosis or pyroptosis are inhibited^13^.

Bacterial pathogens have various bacterial infectious stratagems to modify the autophagic process in manipulating the cell death mechanism. For instance, *S. typhimurium* and *M. tuberculosis* invade host cells and dwell within cytosolic vacuoles for their replication, which is targeted by autophagy for bacterial restriction and host cell survival^14^,^15^. In contrast, *S. aureus* and *L. monocytogenes* have been shown to utilize autophagy for their replication and dissemination in the process of host cell killing^16^,^17^. However, the molecular mechanism of autophagy inducing cell death in the pathogenesis still remains to be clarified. Thus, determining the roles of VvhA in promoting autophagy-related cell death and its regulating strategies can be used in therapeutic applications against *V. vulnificus* infections^18^.

In the circumstance of infection, the intestinal epithelium is capable of maintaining tissue homeostasis by regulating barrier function and immune responses^19^. The *in vitro* Caco-2 cell line model system is biologically compatible with the *in vivo* system because its monolayer exhibits the characteristics of the human intestinal epithelium. The Caco-2 cell culture model was also used in study to evaluate bacterial invasion and pathogenesis^20^,^21^,^22^. In the present study, we investigated the molecular mechanism of VvhA related to autophagy in cell death induced by *V. vulnificus in vitro* and *in vivo*. Thus, we evaluated the functional role of VvhA in the pathogenesis of *V. vulnificus*.

## Results

### rVvhA induced autophagy in promoting cell death

**Figure 1:**
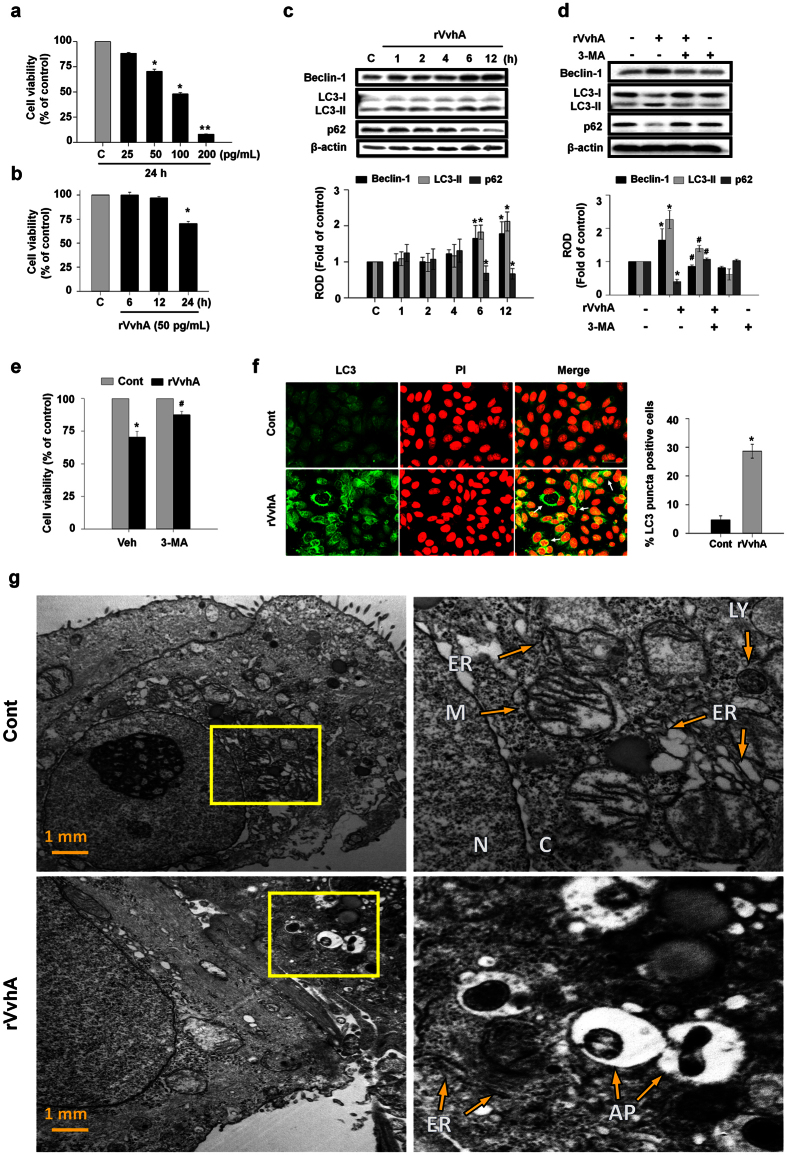
rVvhA induced autophagy in promoting cell death. (**a**) Dose responses of rVvhA for 24 h in MTT assay are shown. Data represent mean ± S.E. n = 4. *P < 0.05, **P < 0.01 versus control. (**b**) Time responses of 50 pg/mL of rVvhA in MTT assay are shown. Data represent mean ± S.E. n = 4. *P < 0.05 versus control. Cells were incubated with 50 pg/mL of rVvhA. (**c**) The effect of rVvhA on expressions of autophagy-related proteins, Beclin-1, LC3, and p62 were determined by western blot. Data represent the mean ± S.E. n = 4. *P < 0.05 versus control. ROD, relative optical density (**d**) Cells were pretreated with a PI3K inhibitor, 3-methyladenine (3-MA, 10 mM) for 60 min prior to rVvhA exposure for 6 h. Selective autophagy markers were analyzed by western blot. Data represent means ± S.E. *P < 0.05 versus control. ^**#**^*P* < 0.05 versus rVvhA alone. (**e**) MTT assay shows that pretreatment with 3-MA improved the cytotoxic effect of rVvhA on Caco-2 cells. Data represent means ± S.E. *P < 0.05 versus vehicle (Veh) alone. ^**#**^*P* < 0.05 versus Veh + rVvhA. (**f**) Autophagosome formation was determined by confocal microscopy using immunofluorescence staining with LC3 (green). Propidium iodide (PI, red) was used for nuclear counter stain (*left panel*). Arrows indicate accumulated LC3-positive autophagosomes in cells. LC3-positive puncta cells were quantified (*right panel*). Data represent the mean ± S.E. *P < 0.05 versus control. (Scale bar = 50 μm) (**g**) Cells were incubated in the presence or absence of 50 pg/mL rVvhA for 6 h. Autophagosome formation was determined by transmission electron microscopy. AP, autophagosome. C, cytosol. ER, endoplasmic reticulum. N, nucleus. M, mitochondria. LY, lysosome.

To determine the functional role of VvhA, Caco-2 cells were treated with recombinant protein (r) VvhA at different concentrations for 24 h. rVvhA induced cytotoxicity in Caco-2 cells from 50 to 200 pg/mL compared to the controls (Fig. 1a). A substantial cytotoxic effect was observed after 24 h of incubation with 50 pg/mL of rVvhA (Fig. 1b). Representative markers of autophagy activation, Beclin-1, microtubule-associated protein 1 light chain 3 (LC3) and p62, were evaluated in cells treated with 50 pg/mL of rVvhA. Expression of Beclin-1 and LC3-II was increased in a time-dependent manner, whereas the level of p62 was attenuated by the treatment with rVvhA (Fig. 1c). To evaluate the functional role of autophagy in rVvhA-induced cell death, we assessed the cell viability of rVvhA-treated Caco-2 cells by pretreating the cells with a PI3K inhibitor, 3-methyladenine (3-MA) for 60 min. 3-MA has been widely used as an autophagy inhibitor^23^. We found that 3-MA significantly inhibited the levels of Beclin-1 and LC3-II and increased the p62 levels (Fig. 1d). Moreover, 3-MA pretreatment effectively rescued the cells from the cytotoxicity induced by rVvhA (Fig. 1e). This result shows that rVvhA induces autophagy activation in promoting cell death. We confirmed that rVvhA increases autophagic vesicle formation through confocal microscopy, in which a significant increase in LC3 puncta positive cells was observed (Fig. 1f). Interestingly, a unique feature of autophagosomes characterized by the peculiar double-membraned vesicle was observed in the cells treated with rVvhA by transmission electron microscopy (TEM) (Fig. 1g). Although there are some swollen mitochondria in a cell treated with rVvhA, we found that there were no statistically significant differences between cells treated with rVvhA and vehicle. Instead, rVvhA caused significant abnormalities like widen endoplasmic reticulum (ER) and autophagic vesicles formation in Caco2 cells (Fig. 1g).

**Figure 2:**
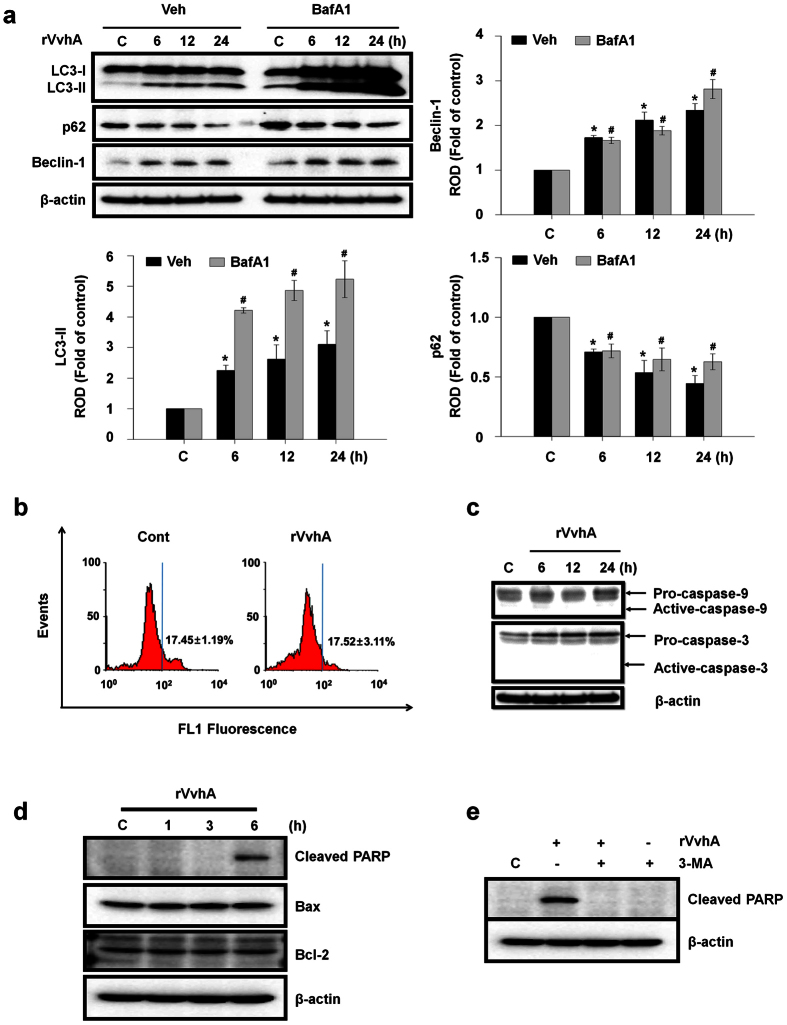
rVvhA induced autophagy-related cell death that are independent of lysosomal membrane permeabilization and caspase activation. (**a**) Cells were incubated in the presence or absence of 10 nM BafA1 for 60 min prior to rVvhA exposure. Time-dependent changes in the level of autophagy markers are shown. Data represent mean ± S.E. n = 3. *P < 0.05 versus Veh alone, ^#^*P* < 0.01 versus BafA1 alone. ROD, relative optical density. (**b**) Lysosomal membrane permeability was evaluated by flow cytometry using the acridine orange staining technique and quantified by the percentage of increase in green fluorescence. Data represent means ± S.E. n = 5. (**c**) Time responses of rVvhA in the activation of Caspase-9 and Caspase-3 were confirmed by western blot with Caspase-9 and Caspase-3 antibodies. n = 3. (**d**) Time responses of rVvhA in the expression of Bax, Bcl-2, and Cleaved PARP were confirmed by western blot. n = 3. (**e**) Cells were incubated in the presence or absence of 3-MA for 60 min prior to rVvhA exposure for 6 h. n = 3.

To confirm the enhanced autophagic flux, we used bafilomycin A1 (BafA1) which is a known inhibitor of V-ATPase (vacuolar-type H^+^ ATPase) that prevents the maturation of autophagic vacuoles^24^. rVvhA significantly enhanced the accumulation of LC3-II in cells pretreated with BafA1, suggesting that the lysosomal degradation pathway was not inhibited by rVvhA (Fig. 2a). We further examined the lysosomal membrane integrity with lysosome targeting dye, acridine orange. Acridine orange has a distinct red fluorescence in the low pH of lysosomes, and emits a green fluorescence when lysosomal membrane ruptures^25^. Interestingly, we found that rVvhA was not able to change the red fluorescence to the green, indicating that the integrity of the lysosomal membrane was not affected by rVvhA (Fig. 2b). We next determined whether rVvhA-induced cell death is related to caspase activation. Activation of Capase-9 and Caspase-3 was not observed after exposure to rVvhA over time (Fig. 2c). However, rVvhA significantly increased the level of cleaved poly(ADP-ribose) polymerase (PARP), a nuclear enzyme activated by DNA strand breaks as a DNA damage marker, but did not show any effect on the expressions of Bcl-2 and Bcl-2-associated X protein (Bax) that regulate mitochondrial dysfunction (Fig. 2d). In addition, autophagy inhibitor, 3-MA significantly inhibited the levels of cleaved PARP (Fig. 2e). Taken together, our data confirmed that rVvhA induces autophagy-related DNA damage, which is not affected by lysosomal/mitochondrial dysfunction and caspase activation.

### Essential role of c-Src in lipid raft-mediated rVvhA signaling

**Figure 3:**
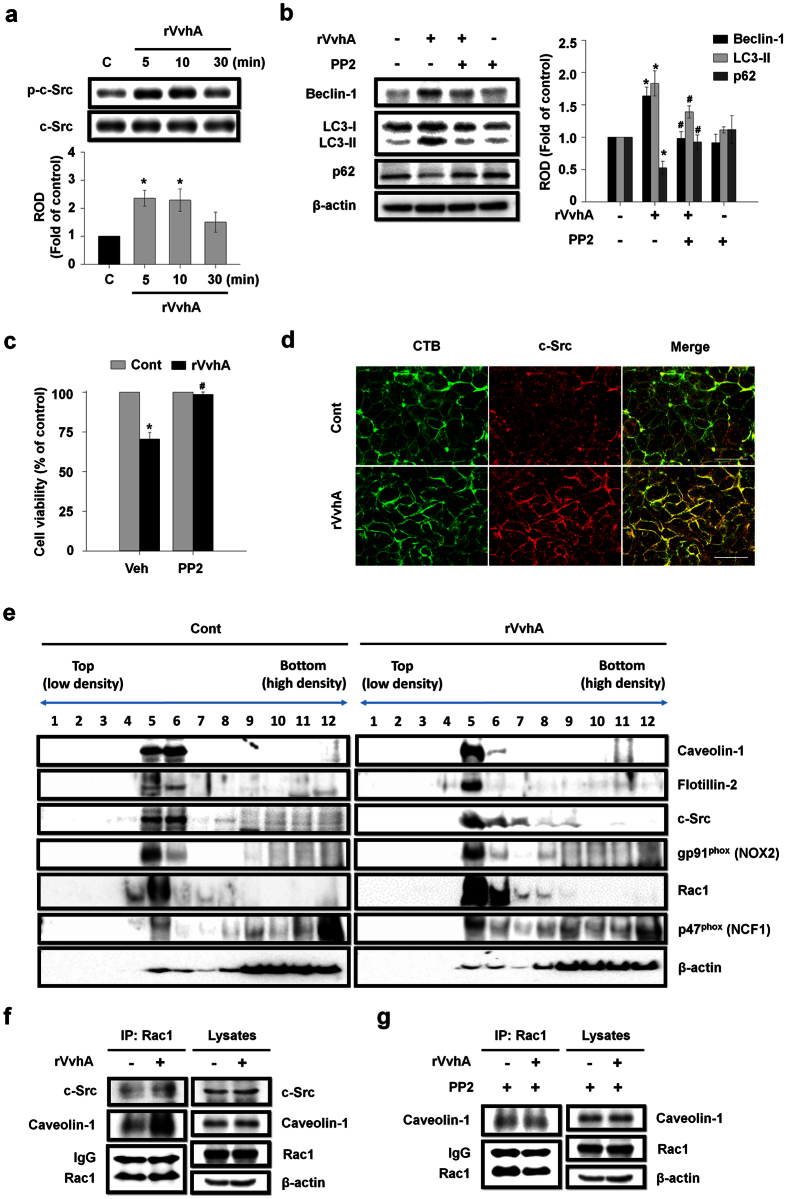
Essential role of c-Src in lipid raft-mediated rVvhA signaling. (**a**) Time responses of rVvhA in c-Src phosphorylation is shown. n = 3. *P < 0.05 versus control. ROD, relative optical density. (**b**) Cells were pretreated with a c-Src inhibitor, PP2 (1 μM) for 60 min prior to rVvhA exposure. Changes in expression of autophagy markers are shown. Data represent mean ± S.E. n = 4. *P < 0.05 versus control. ^**#**^*P* < 0.05 versus rVvhA alone. (**c**) The effect of PP2 pretreatment on rVvhA-induced cytotoxicity was determined by MTT assays. Data represent mean ± S.E. n = 4. *P < 0.05 versus control. ^**#**^*P* < 0.05 versus Veh + rVvhA. (**d**) The increased co-localization of CTB (green) and c-Src (red) was determined by confocal microscopy using immunofluorescence staining. Scale bar = 100 μm. n = 4. (**e**) Membrane lipid raft fractions were prepared by discontinuous sucrose density gradient fractionation, and the location of Caveolin-1, Flotillin-2, c-Src, gp91^phox^ (NOX2), Rac1, and p47^phox^ (NCF1) was determined by western blot. n = 3. (**f**) Rac1 co-immunoprecipitated with c-Src and Caveolin-1 is shown (*left panel*). Expression of c-Src, Caveolin-1, and Rac1 in total cell lysates is shown (*right panel*). (**g**) PP2 inhibited the interaction of Rac1 with Caveolin-1.

It has long been suggested that Src tyrosine kinases play a role in the host defense to bacterial infection^26^,^27^,^28^. c-Src is enriched in lipid rafts and regulates numerous signals^29^,^30^. To investigate the role of tyrosine kinase c-Src in rVvhA-stimulated cellular responses, we examined the levels of c-Src phosphorylation in cells treated with rVvhA. The phosphorylation of c-Src in the cells treated with rVvhA increased from 5 min and slightly recovered at 30 min (Fig. 3a). We found that a c-Src inhibitor, PP2, significantly inhibited the autophagy activation by rVvhA in Caco-2 cells (Fig. 3b). Moreover, the cytotoxic effect of rVvhA in Caco-2 cells was decreased with pretreatment of PP2 (Fig. 3c). To determine the role of rVvhA in the interaction of c-Src with lipid rafts, we did confocal microscopy. Cholesterol-rich membrane lipid rafts can be visualized by confocal microscopy using fluorescence immunostaining with cholera toxin B (CTB)^31^. CTB showed that rVvhA increased the interaction of c-Src with the lipid rafts (Fig. 3d).

We then determined how c-Src mediates the rVvhA-induced signals within the lipid rafts using sucrose gradient centrifugation (Fig. 3e). The lipid raft markers Caveolin-1 and Flotillin-2 were found in fractions 5–6 at basal levels. Additionally, we found that c-Src, gp91^phox^ (NOX2), p47^phox^ (NCF1) and Rac1 were mainly localized in fractions 5–6, suggesting that c-Src is co-localized with the NADPH oxidase (NOX) complex in the lipid rafts. However, the location of Caveolin-1, Flotillin-2, c-Src, and Rac1, and NADPH oxidase complex are changed and aggregated into fraction 5. Since above approaches are qualitative at best, we further tried to quantify the results by using co-immunoprecipitation of Rac1 with proteins related to the lipid rafts in the presence of rVvhA (Fig. 3f). The lysates in right panel of Fig. 3f are consistent with the input before putting them in gradient and represent the expression levels of proteins are not changed by rVvhA treatment. Instead, we found that Rac1 co-immunoprecipitated with c-Src and caveolin-1, and importantly, that these interactions were significantly enhanced by the rVvhA treatment. This means that the locations of Rac1 and c-Src are changed and aggregated within lipid raft without changes their expression levels by treatment of rVvhA. In addition, the interaction of Rac1 with Caveolin-1 induced by rVvhA was significantly attenuated by pretreatment with the c-Src inhibitor PP2 (Fig. 3g). These results support that c-Src mediates the raft-dependent rVvhA signaling pathway by increasing the interaction between lipid raft and the NOX complex.

### Phosphorylation of ERK and eIF2α by NOX-mediated ROS production is required for rVvhA-induced autophagy activation

**Figure 4:**
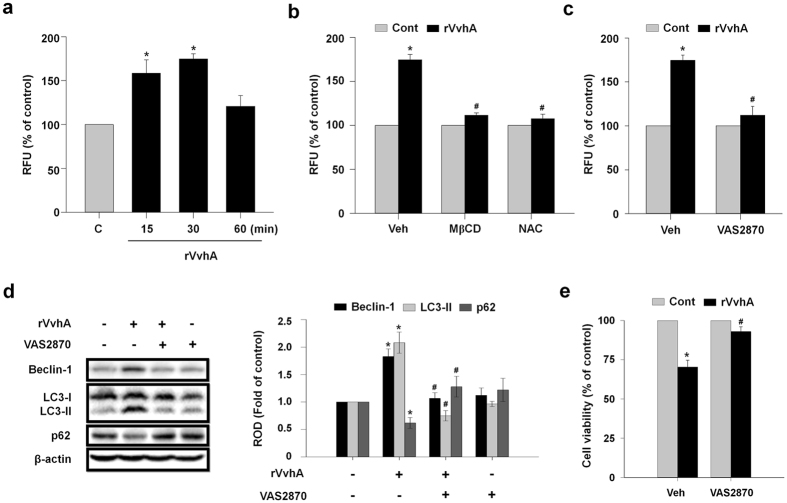
Requirement of NOX-mediated ROS production in rVvhA-induced autophagy activation. (**a**) Time responses of rVvhA in ROS production are shown. n = 5. *P < 0.05 versus control. RFU, relative fluorescence units. (**b**) Cells were pretreated with MβCD (0.1 mM) and NAC (1 mM) for 60 min prior to rVvhA (50 pg/mL) exposure for 30 min. The level of ROS production is shown. Data represent mean ± S.E. n = 4. *P < 0.05 versus Veh alone. ^**#**^*P* < 0.05 versus Veh + rVvhA. (**c**) Cells were preincubated with VAS2870 (2 μM) for 60 min prior to rVvhA exposure. The effect of VAS870 on ROS production of cells treated with rVvhA. Data represent mean ± S.E. n = 4. *P < 0.05 versus Veh alone. ^**#**^*P* < 0.05 versus Veh + rVvhA. (**d**) The effect of VAS2870 on changes of the expression level of autophagy-related proteins in cells treated with rVvhA. Data represent mean ± S.E. n = 4. *P < 0.05 versus control. ^**#**^*P* < 0.05 versus Veh + rVvhA. (**e**) Cells were pretreated with VAS2870 for 60 min prior to rVvhA exposure for 24 h. Cell viability was determined by MTT assay. Data represent mean ± S.E. n = 4. *P < 0.05 versus Veh alone. ^**#**^*P* < 0.05 versus Veh + rVvhA alone.

rVvhA significantly increased the ROS production between 15 and 30 min (Fig. 4a), which was inhibited by pretreatment with a lipid raft destructor methyl-β-cyclodextrin (MβCD) and a ROS scavenger N-acetylcysteine (NAC) (Fig. 4b). To determine the source of ROS produced by rVvhA, Caco-2 cells were treated with a NOX inhibitor, VAS2870, for 60 min prior to exposure to rVvhA. VAS2870 significantly inhibited the ROS production induced by rVvhA (Fig. 4c). These observations suggest that the ROS production of rVvhA is dependent on lipid raft-mediated NOX signaling. The autophagy activation and cytotoxicity induced by rVvhA were also significantly blocked by VAS2870 pretreatment (Fig. 4d,e).

**Figure 5:**
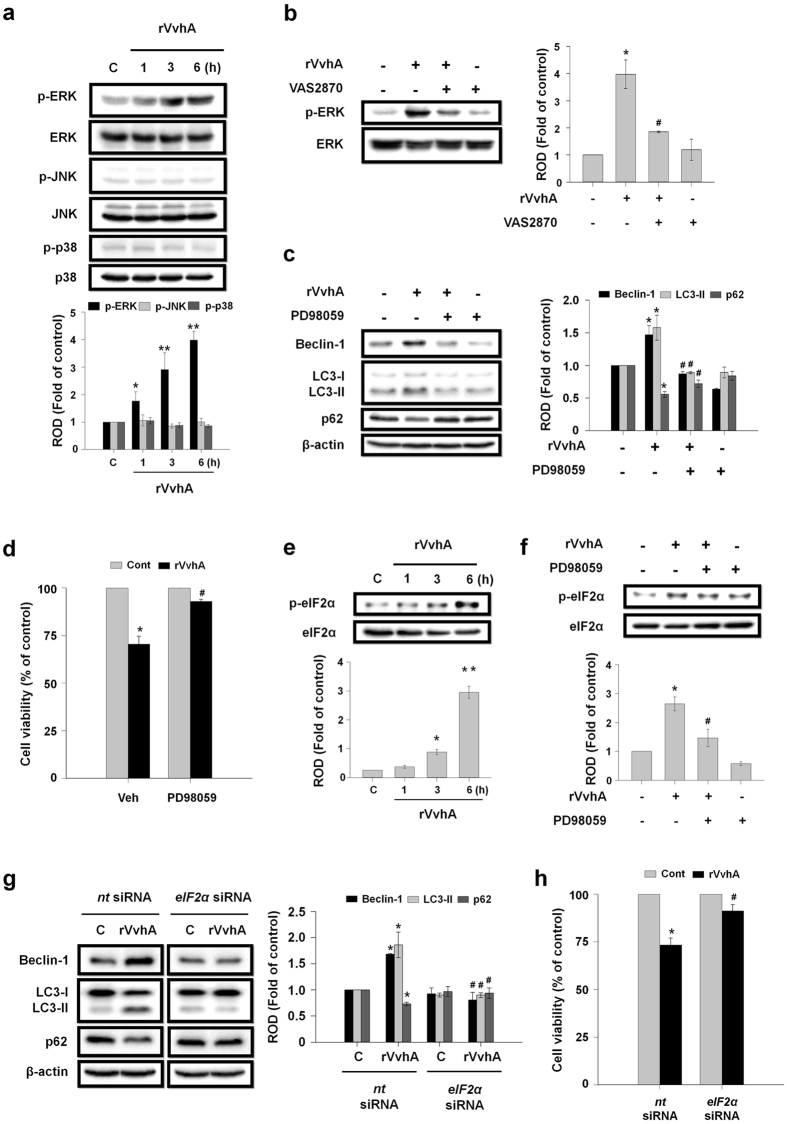
Phosphorylation of ERK by ROS is required of eIF2α phosphorylation and autophagy induction. (**a**) Caco-2 cells were incubated in the presence of rVvhA (50 pg/mL) for various times (0–6 h) and then harvested. Total protein was extracted and blotted with p-ERK, p-JNK, p-p38, ERK, JNK, and p38 antibodies. Data represent mean ± S.E. n = 4. *P < 0.05; **P < 0.01 versus control. (**b**) Cells were pretreated with VAS2870 for 60 min prior to rVvhA exposure for 3 h. The phosphorylation of ERK was determined by western blotting with p-ERK and ERK antibodies. n = 4. *P < 0.01 versus control. ^**#**^*P* < 0.05 versus rVvhA alone. (**c**) Cells were pretreated with an ERK inhibitor, PD98059 (10 μM) for 60 min prior to rVvhA exposure for 6 h. Changes in expression level of autophagy markers are shown. Data represent mean ± S.E. n = 4. *P < 0.05 versus control. ^**#**^*P* < 0.05 versus rVvhA alone. (**d**) Cell viability was determined by MTT assay. Data represent mean ± S.E. n = 4. *P < 0.05 versus control. ^**#**^*P* < 0.05 versus Veh + rVvhA. (**e**) Phosphorylation of eIF2α in cells treated with rVvhA is shown. Data represent mean ± S.E. n = 4. *P < 0.05; **P < 0.01 versus control. (**f**) Cells were pretreated with PD98059 for 60 min prior to rVvhA exposure. Phosphorylation of eIF2α was determined by western blot. *P < 0.05 versus control. ^**#**^*P* < 0.05 versus rVvhA alone. Cells were transfected with *eIF2α* siRNA or nontargeting (*nt*) siRNA for 36 h prior to rVvhA exposure. Knockdown of eIF2α with siRNA decreased rVvhA-induced autophagy activation (**g**) and cytotoxicity (**h**). Data represent mean ± S.E. n = 4. *P < 0.05 versus *nt* siRNA alone. ^**#**^*P* < 0.05 versus *nt* siRNA + rVvhA.

Many authors have shown that mitogen-activated protein kinases (MAPKs) mediate integrated stress response and regulate autophagy^32^,^33^,^34^. We explored the role of MAPK in the process of NOX-dependent autophagy induction. In our results, the phosphorylation of ERK in Caco-2 cells was significantly increased by exposure to rVvhA in a time-dependent manner, while the phosphorylation of JNK and p38 was not affected (Fig. 5a). Increased phosphorylation of ERK was significantly inhibited by the NOX inhibitor VAS2870 (Fig. 5b). These results suggest that ERK plays an important role in the NOX-mediated ROS signaling pathway. Furthermore, an ERK inhibitor, PD98059 significantly inhibited the lipidation of LC3 (Fig. 5c) as well as the cytotoxicity (Fig. 5d) that are both induced by rVvhA. Many authors reported that eIF2α is involved in ERK-mediated autophagy induction^35^,^36^,^37^. rVvhA stimulated the phosphorylation of eIF2α which was inhibited by PD98059 pretreatment, suggesting that ERK plays an important role in eIF2α activation (Fig. 5e,f). Additionally, we found that rVvhA significantly induces CCAAT/enhancer binding protein (C/EBP) homologous protein (CHOP) (Supplementary Fig. 1a), which is one of the components of the ER stress-mediated cell death pathway, and the expression of CHOP was inhibited by transfection with siRNA for *eIF2α* (Supplementary Fig. 1b), indicating that CHOP is a downstream target of eIF2α phosphorylation and ER stress is also involved in rVvhA-induced autophagic cell death possibly trough ROS production^38^. Interestingly, we found that knockdown of eIF2α by siRNA inhibits the effect of rVvhA on autophagy activation and cytotoxicity (Fig. 5g,h). Therefore, the results indicate that the NOX-mediated ROS production by rVvhA induces the phosphorylation of ERK and eIF2α which is required for autophagy activation.

### Phosphorylation of eIF2α is crucial in rVvhA-induced autophagic cell death

**Figure 6:**
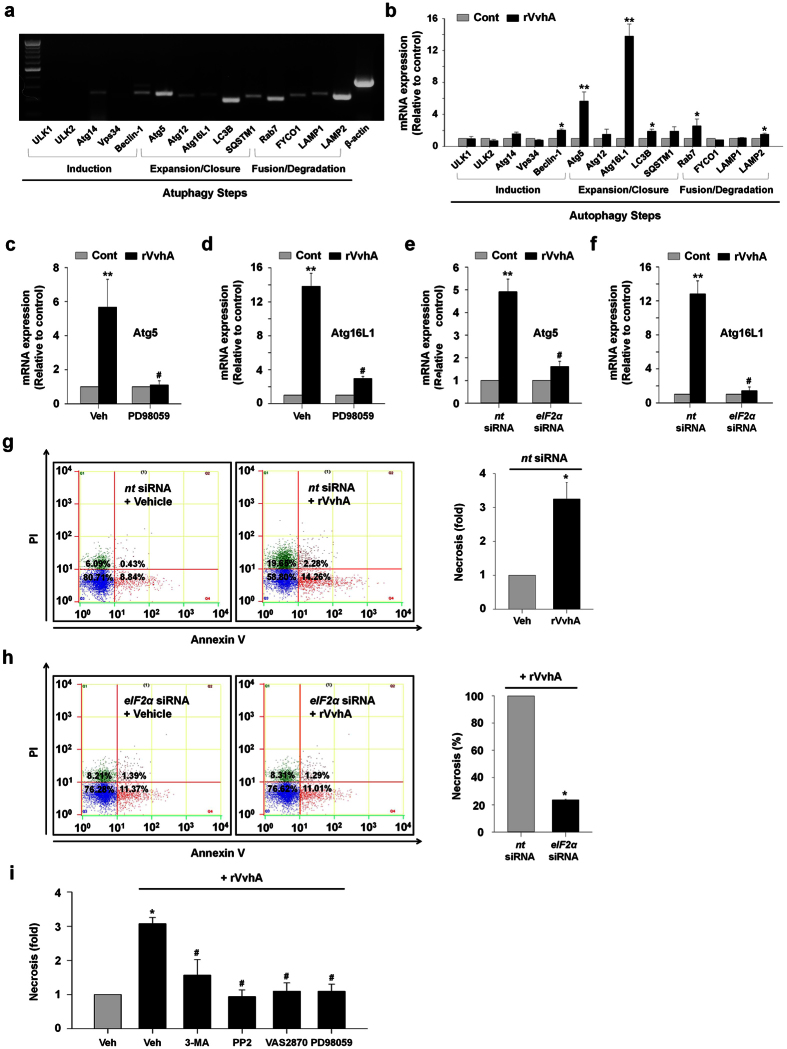
Phosphorylation of eIF2α is crucial in rVvhA-induced autophagic cell death. At 3 h after rVvhA (50 pg/mL) exposure, total RNA of cells were extracted. (**a**) RT-PCR results show mRNA expression of autophagy-related proteins in Caco-2 cells. n = 3. (**b**) The effect of rVvhA on the expression of autophagy-related proteins was evaluated by qRT-PCR. The expression level of Atg5 and Atg16L1 was markedly increased by rVvhA treatment in cells. n = 4. *P < 0.05; **P < 0.01 versus control. Cells were incubated with PD98059 for 60 min prior to rVvhA exposure. The mRNA expression of Atg5 (**c**) and Atg16L1 (**d**) are shown. Data represents mean ± S.E. n = 4. *P < 0.01 versus control. ^**#**^*P* < 0.01 versus Veh plus rVvhA. Cells were transfected with *eIF2α* siRNA or non-targeting (*nt*) siRNA for 36 h prior to rVvhA exposure. Knockdown of eIF2α with siRNA silenced the increases of mRNA expression of Atg5 (**e**) and Atg16L1 (**f**) by rVvhA. Data represent mean ± S.E. n = 4. *P < 0.01 versus *nt* siRNA alone. ^**#**^*P* < 0.01 versus *nt* siRNA + rVvhA. Cells were incubated with rVvhA (50 pg/mL) for 24 h. (**g**) Percentages of necrosis, survival, and apoptosis were measured by using PI/Annexin V dual staining and flow cytometry (*left panel*). Quantitative analysis of necrotic cells (Q1) by FACS analysis is shown (*right panel*). (**h**) Dual stain of Annexin V and PI shows that the necrotic (Q1) cell death was increased by rVvhA, which was reduced by knockdown of eIF2α with siRNA. n = 3. (**i**) Cells were preincubated with 3-MA, PP2, VAS2870, and PD98059 for 1 h prior to rVvhA exposure. Quantitative analysis of the percentage of necrotic cells (Q1) by FACS analysis is shown. n = 3.

To determine the expression levels of autophagy-related proteins, we performed reverse transcription polymerase chain reaction (RT-PCR). The results show that Caco-2 cells strongly expressed the mRNAs of Atg5, Atg16L1, LC3B, Rab7 and Lamp2; however, the expression levels of other proteins were low or not detected (Fig. 6a). Using quantitative real-time polymerase chain reaction (qRT-PCR), we found that rVvhA specifically increased the mRNA expressions of Atg5 and Atg16L1 (Fig. 6b). Atg5 and Atg16L1 are required for the formation of autophagosomes^9^. Interestingly, PD98059 markedly inhibited the levels of Atg5 and Atg16L1 evoked by rVvhA in Caco-2 cells (Fig. 6c,d). In addition, knockdown of eIF2α by siRNA effectively blocked the stimulating effect of rVvhA on the expression of Atg5 and Atg16L1 (Fig. 6e,f). These results suggest that ERK-mediated eIF2α phosphorylation is essential for autophagosome formation. We performed flow cytometric analysis using propidium iodide (PI) and Annexin V for identification of necrotic and apoptotic cells caused by rVvhA treatment (Fig. 6g). rVvhA significantly induced necrotic cell death in Caco-2 cells compared to the control while apoptotic cell death was not evident. We confirmed that the necrotic cell death induced by rVvhA was significantly attenuated by knockdown of eIF2α by siRNA (Fig. 6h). In parallel, the rVvhA-induced necrotic cell death was significantly decreased by pretreatment with 3-MA, PP2, VAS2870, and PD98059 (Fig. 6i). These data provide important evidence that rVvhA regulates autophagy through ERK/eIF2α phosphorylation during autophagic cell death in intestinal epithelial cells.

### VvhA mediates intestinal inflammation and autophagy induced by *V. vulnificus*

**Figure 7:**
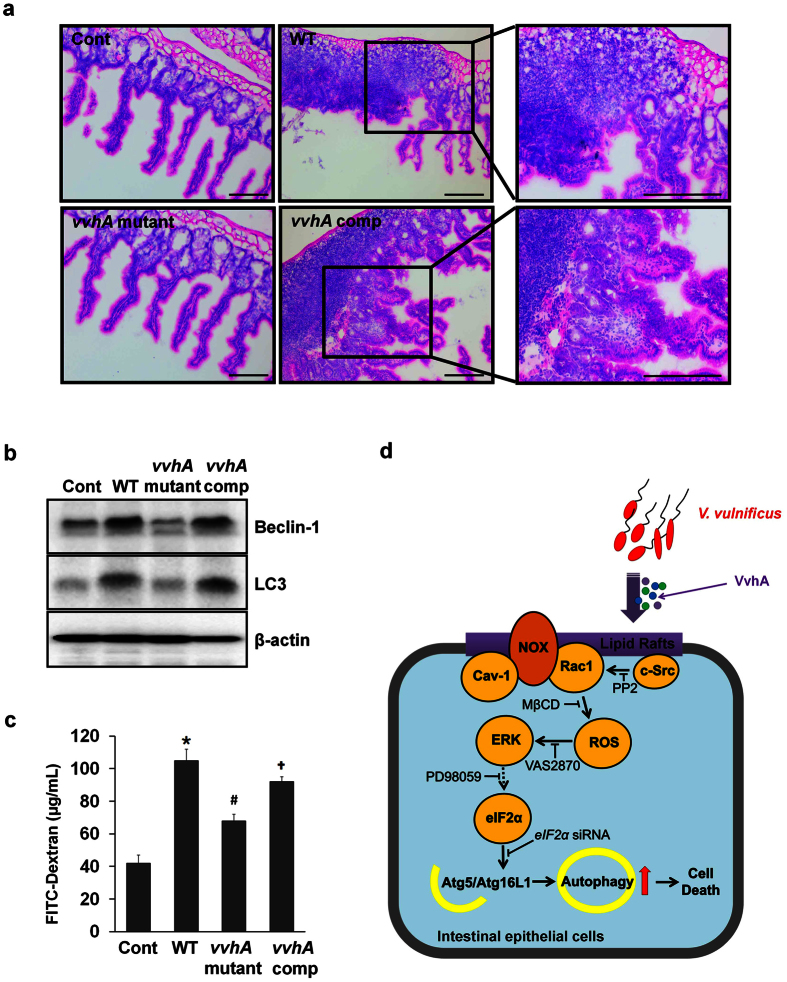
VvhA mediates intestinal inflammation and autophagy induced by *V. vulinficus.* ICR mice were inoculated intragastrically with WT, boiled WT (Cont), *vvhA* mutant, and *vvhA* complement (comp) at 1.1 × 10^9^ CFU/mL for 18 h. n = 7. (**a**) Representative images of H&E stained ileum tissues are shown. Damaged intestinal tissue is shown at higher magnification. Scale bar represents 100 μm. (**b**) The expression levels of autophagy markers in ileum tissue were determined by western blot. (**c**) Mice received intragastric inoculation of Cont, WT, *vvhA* mutant, and *vvhA* comp at 1.1 × 10^9^ CFU/mL, and were sacrificed 18 h later. The FITC-dextran was orally administered into mice for 4 h before euthanasia. Analysis of FITC-dextran translocation into the blood stream by luminometry reveals intestinal paracellular permeability. n = 7. (**d**) A proposed model for VvhA-evoked signaling pathway in intestinal epithelial cells. VvhA plays an essential role in the dissemination and pathogenesis of *V. vulnificus* in intestinal cells, where VvhA induces autophagy-related cell death through lipid raft-mediated c-Src/NOX signaling pathway and ERK/eIF2a-dependent autophagy activation.

To evaluate the functional role of *V. vulnificus* VvhA, seven-week-old ICR mice received intragastric administration of boiled *V. vulnificus* (Cont), *V. vulnificus* (WT), *V. vulnificus (vvhA* mutant), a mutant strain deficient in the *vvhA* gene and *V. vulnificus (vvhA* comp), the *vvhA* mutant strain complemented with the functional *vvhA* gene, at 1.1 × 10^9^ CFU/mL. We euthanized the mice 18 h after the inoculation. We found that WT strongly induces the injury of intestinal epithelium by shortening the length of villi compared to the control (Fig. 7a). Interestingly, the damage effect of *V. vulnificus* was not observed in the intestine treated with the *vvhA* mutant. In contrast, the mice infected with the *vvhA* comp showed completely restored injury responses like that seen in the WT. On the other hand, the infection of mice with the WT showed elevated expression of autophagy-related proteins, Beclin-1 and LC3, which was prevented by infection with the *vvhA* mutant (Fig. 7b). Moreover, the expression of autophagy markers after infection by the *vvhA* comp was identical to that of the WT. Therefore, these results suggest that VvhA is a crucial virulence factor responsible for inflammation as well as autophagy activation induced by *V.vulnificus*. We further investigated the activity of VvhA on the paracellular permeability of intestinal epithelial cells which promotes the invasion of *V. vulnificus*. ICR mice were inoculated intragastrically with the Cont, WT, *vvhA* mutant, and *vvhA* comp strains at 1.1 × 10^9^ CFU/mL and then were given oral administration of FITC-labeled 4kDa dextran for 4 h before sacrifice to estimate the intestinal leakage and translocation of FITC-dextran to the blood stream. WT significantly increased the serum level of FITC-dextran compared to the control (Fig. 7c). However, the increased level of FITC-dextran by WT was reduced by the *vvhA* mutant. Interestingly, *vvhA* comp reinstalled the ability of paracellular permeabilization. These results suggested that VvhA has ability to induce the dissemination of *V. vulnifcus* into blood stream.

## Discussion

Our results clearly show here that rVvhA upregulates autophagy flux through c-Src-mediated ROS production and ERK/eIF2*α* phosphorylation in promoting the necrotic cell death of intestinal epithelial cells. To date, this is the first study to provide evidence on the pathogenic mechanism of VvhA produced by *V. vulnificus*, which kills host cells via autophagic cell death, and to reveal novel therapeutic targets for *V. vulnificus* infection. In contrast to our data, previous results revealed that cytolysin, a VvhA homologous protease produced by *V. cholerae*, actually inhibited autophagy in inducing host cell death^39^. However, this study did not define whether the lower autophagy rate was simply explained by the consequence of severe cell death induced by cytolysin because it has been well characterized that cell death induced by lysosomal permeabilization and/or caspase activation is also closely associated with impairment of the autophagic process. Given that the autophagic cell death induced by rVvhA did not affected by lysosomal permeabilization, mitochondrial dysfunction, and caspase activation during autophagic cell death, we suggest for the first time that rVvhA may have unique autophagy signaling pathways. These results are further supported by previous reports showing that *M. tuberculosis* and *S. aureus* induce caspase-independent cell death, although lysosomal dysfunction was the ultimate cause leading to host cell death^16^,^40^. Thus, our results strongly suggest that *V. vulnificus* triggers host cell death by producing VvhA with a unique mode of action in contrast to other pathogenic bacteria.

We subsequently have shown that rVvhA induces rapid activation of c-Src in promoting autophagic cell death. The importance of the c-Src signaling pathway in autophagy induction is well underscored by the finding that autophagic cell death induced by a cell-permeable pan-caspase peptide inhibitor requires c-Src activation^41^. These findings are consistent with the results showing that bacterial signals induced by *S. flexneri* and *C. perfringens* stimulate c-Src activation in promoting caspase-independent host cell death^42^,^43^. Several studies have indicated that c-Src associates with the lipid rafts which acts as a hub linking signals between the inner and outer environments of cells^29^,^30^. In the present study, we found that rVvhA evokes the rearrangement of lipid rafts with the aggregation of c-Src and that the silencing of c-Src significantly inhibits autophagic cell death. This is further supported by previous results which showed that cytolysin secreted by *V. vulnificus* binds to host membrane lipid rafts and forms transmembrane pore to transfer its bacterial signals into host cells^44^,^45^,^46^. Together, our data indicate that c-Src is a major mediator of autophagic cell death triggered by rVvhA within host membrane lipid rafts.

Lipid rafts are also another important element in the initiation of ROS production^29^. In the present study, we further found that c-Src activated by rVvhA significantly regulates the clustering of the redox signaling platform through gp91^phox^ (NOX2) coupled with cytosolic factors that include p47^phox^ (NCF1), p67^phox^ (NCF1), and small GTPase Rac1 in the lipid rafts and that these processes subsequently stimulate intracellular ROS production. Importantly, the inhibition of NOX2 clearly attenuated the autophagic cell death induced by rVvhA. This means that c-Src acts to transduce ROS signals via aggregation of NOX components that are responsible for autophagic cell death. Supporting our finding, previous data revealed that *S. typhimurium* infection also resulted in NOX2-mediated ROS production, although NOX activity is required for autophagy promoting host cell survival^47^. ROS generated by a bacterial infection has been shown to induce oxidative inactivation of several proteins harboring oxidant-sensitive thiol groups, thereby activating many redox-sensitive proteins which include regulators of MAP kinase pathways^48^. Interestingly, rVvhA uniquely induced autophagic cell death through the phosphorylation of a distinct MAP kinase, ERK. A previous report showed that *S. aureus* induces autophagy through phosphorylation of JNK but not p38 MAPK and ERK in macrophages^49^. This differs from our data in that rVvhA increases ERK phosphorylation through NOX2-mediated ROS production. In addition, *M. tuberculosis* and *E. coli* were shown to modulate autophagy system through the ROS-mediated p38MAPK signaling pathway^50^,^51^. These data collectively indicate that rVvhA selectively regulates specific MAPK phosphorylation during autophagic cell death.

Previous work showed that the eukaryotic translation initiation factor 2 (eIF2) is critically involved in the autophagic process and translational arrest during nutrient deprivation and virus infection^37^. In the present study, we found that rVvhA significantly regulates ERK-mediated phosphorylation of eIF2α. It is not clear whether the regulatory effect of rVvhA in promoting the phosphorylation of eIF2α is a direct sequential result of the ERK phosphorylation or alternatively, an indirect process involving other translational signaling events. However, it was clearly shown that ERK phosphorylation induced by amino acid deprivation stimulates the binding of uncharged tRNA to activate general control nonderepressible-2 (GCN2) kinase which phosphorylates serine 51 of eIF2α^36^. Importantly, our data revealed that the activation of eIF2α induced by rVvhA distinctively regulates the expression of Atg5 and Atg16L1, which are required for autophagosome formation. Atg5 covalently conjugates with Atg12 and interacts with Atg16L1 to form a dimeric complex called the Atg16L1 complex which promotes LC3 lipidation and correct localization on expanding phagophores for elongation of the autophagic membrane^52^,^53^. Thus, these results indicate that rVvhA induces the formation of autophagosomes via ERK-eIF2α signaling pathway in promoting the intestinal epithelial cell death. In keeping with the unique signaling pathway of rVvhA triggering autophagic cell death, our *in vivo* study showed that VvhA is an important virulence factor secreted by *V. vulnificus* responsible for intestinal autophagy induction and a severe inflammatory response which stimulates LC3 and beclin-1 expression. Similarly, a previous study showed that the autophagy pathway plays a critical role in the control of inflammatory signaling through the stress-responsive transcription factor as well as the inflammasome^54^. Although the autophagy pathway may prevent tissue inflammation through its role in apoptotic corpse clearance, our data present the possibility that VvhA influences the inflammatory pathogenesis of *V. vulnificus* by regulating autophagic cell death without the apoptotic process mediated by caspases and mitochondrial dysfunctions.

Collectively, the results of this study suggest that rVvhA is responsible for the pathogenesis of *V. vulnificus* by autophagy upregulation, through which rVvhA stimulates the c-Src-mediated interaction of lipid raft molecules with the NOX complex to activate ERK/eIF2α, which is essential for the Atg5/Atg16L1-dependent autophagy induction (Fig. 7d). Thus, highlighting the signaling pathways involved in the rVvhA-stimulated autophagic cell death pathway may provide potential therapeutic targets for strategic modulations of *V. vulnificus* infections.

## Materials and Methods

### Chemicals

Dulbecco’s Modified Eagle Medium (DMEM) and FBS were purchased from GE Healthcare (Logan, UT, USA). The following antibodies were purchased: LC3 and p62 antibodies (Novus Biologicals, Littleton, CO, USA); Bax, Beclin-1, Bcl-2, Caspase-9, Caspase-3, Caveolin-1, CHOP, cleaved PARP, Flotillin-2, c-Src, ERK, p-ERK, JNK, p-JNK, p38, and p-p38 antibodies (Santa Cruz Biotechnology, Paso Robles, CA, USA); gp91^phox^ and Rac1 antibodies (BD Biosciences, Franklin Lakes, NJ, USA); p47^phox^ antibody (LifeSpan Biosciences, Seattle, WA, USA); eIF2α and p-eIF2α antibodies (Cell Signaling Technology, Danvers, MA, USA); HRP-conjugated goat anti-rabbit/mouse IgG antibodies (Jackson Immunoresearch, West Grove, PA, USA). Rapamycin, 3-methyladenine, bafilomycin A1, PP2, methyl-β-cyclodextrin, N-acetylcysteine, VAS2870, and PD98059 were purchased from Sigma Aldrich (St. Louis, MO, USA). 2′, 7′-dichlorofluorescein diacetate (CM-H_2_DCFDA) was obtained from Invitrogen (Carlsbad, CA, USA). All other reagents were of the highest purity commercially available.

### Cell culture and viability test

Caco-2 cells were purchased from American Type Culture Collection (Manassas, VA, USA) and were grown at 37 °C in 5% CO_2_ in DMEM with 4 mM L-glutamine, complemented with 10% FBS and antibiotics (10 units/mL penicillin G and 10 μg/mL streptomycin). For the experiments, cells were seeded on culture plate at 3 × 10^5^ cells/cm^2^ and maintained until confluent. Cell viability was measured using MTT assay as described previously^45^. Briefly, cells were pretreated with indicated inhibitors before being exposed to rVvhA. MTT solution (final concentration, 1 mg/mL) was added to each well, and the plates were incubated at 37 °C for 4 h. The formazan crystal was dissolved with 150 μL of DMSO and analyzed using a spectrophotometer.

### Purification of the recombinant haemolysin (r) VvhA

rVvhA was prepared as described previously^45^. Briefly, the coding region of *vvhA* was amplified and then cloned into a His6-tag expression vector, pET29a(+) (Novagen, Madison, WI, USA), resulting in pKS1201 (Supplementary Table 1). *E. coli* BL21 (DE3) carrying the pKS1201 was grown in LB-ampicillin media and treated with 1 mM IPTG for inducing protein expression. The cells were harvested and prepared for isolating the soluble fraction. Cell lysate was mixed with 1 mL of Ni-NTA agarose (Qiagen, Valencia, CA) and loaded on Bio-Spin Chromatography Columns (Bio-Rad Laboratories, Hercules, CA, USA). After washing columns with buffer A, the bound VvhBA protein was eluted with buffer A containing 300 mM imidazole. Purified rVvhA was dialyzed using Slide-A-Lyzer Dialysis Cassettes (Thermo Scientific, Hudson, NH, USA).

### Immunofluorescence analysis and transmission electron microscopy (TEM)

Cells were fixed with 80% acetone for 10 min, permeabilized in 0.1% Triton X-100 in PBS for 10 min, and blocked in PBS containing 5% (v/v) normal goat serum for 30 min at room temperature. Samples were then incubated with primary antibody for overnight at 4 °C followed by staining with Alexa 488-conjugated goat anti-rabbit IgM (Invitrogen, Carlsbad, CA, USA), and counterstained with propidium iodide (PI) for 2 h. Samples were imaged by Olympus FluoView™ 300 confocal microscope. For transmission electron microscopy, rVvhA treated cells were fixed in Karnovsky’s solution and 2% OsO_4_ in 0.1 M cacodylate at 4 °C for 2 hours, respectively. The cells were rinsed with distilled water briefly and stained with 0.5% uranyl acetate en bloc solution for overnight. After dehydration in graded ethanol series, samples were infiltrated with Spurr’s resin. The samples were imaged by JEM1010 (JEOL, Tokyo, Japan) transmission electron microscope operating at 80 kV.

### Western blot and Immunoprecipitation analysis

Cells were lysed with RIPA lysis buffer containing 1 mM protease inhibitor cocktail (Thermo Fisher Scientific, Rockford, IL, USA). Cell lysates were separated by SDS-PAGE and transferred onto polyvinylidene difluoride (PVDF) membrane. Membranes were incubated with indicated antibody in TBST followed by ECL treatment for detecting signals. For the immunoprecipitation analysis, 1 mg of cell lysates was mixed with each antibodies (10 μg) and incubated for 4 h. Protein A/G PLUS-agarose (Santa Cruz Biotechnology, Paso Robles, CA, USA) was added to the mixture which was incubated for an additional 12 h. After washing, the beads were boiled in sample buffer for releasing binding proteins. Samples were analyzed by immunoblotting.

### Flow cytometry

The necrotic cell death was evaluated with an Annexin V and PI staining kit (BD Biosciences, Franklin Lakes, NJ, USA) according to the manufacturer’s instructions. Briefly, single cell suspension was prepared with Annexin V binding buffer at 1 × 10^5^ cells and stained with Annexin V (25 μg/mL) and PI (125 ng/mL) for 15 min at room temperature in the dark. The sample was examined by flow cytometry and analyzed using CXP software (Beckman Coulter, Brea, CA).

### Assessment of lysosomal membrane permeability

Cells were incubated with 5 μM acridine orange (AO) to label lysosomes (Sigma, St. Louis, MO, USA) for 15 min at 37 °C and washed twice with PBS prior to rVvhA exposure. AO displays red fluorescence when highly concentrated in acidic lysosomes and green fluorescence when outside the lysosomes. The increase in green fluorescence due to the release of AO from ruptured lysosomes was examined by flow cytometry in the FL1 channel.

### Fractionation of detergent-resistant membrane lipid raft

Cells were washed with cold PBS, scraped into 2 mL of 500 mM Na_2_CO_3_ (pH 11.0), and homogenized with a Sonicator 250 apparatus (Branson Ultrasonic, Danbury, CT). A discontinuous sucrose gradient (5, 35 and 45%) was formed and centrifuged at 40,000 × g for 20 h in a Beckman SW41 Rotor (Beckman Coulter, Fullerton, CA). Samples were divided to twelve fractions and analyzed by immunoblotting.

### Reactive oxygen species (ROS) measurement

The intracellular ROS levels were measured by fluorometry using the intracelluar oxidation of CM-H_2_DCFDA. The rVvhA-treated cells were incubated with 10 μM H_2_DCFDA for 30 min. After washing with PBS, the DCF fluorescence of the cells was examined using a luminometer (Victor3; Perkin-Elmer, MA, USA) at excitation and emission wavelengths of 485 and 535 nm, respectively.

### Reverse transcription and quantitative real-time PCR

Total RNA was extracted using the MiniBEST Universal RNA Extraction Kit (Takara Bio, Shiga, Japan). A reverse transcription (RT) was carried out with 1 μg of total RNA using a Maxime RT premix kit (iNtRON Biotechnology, Sungnam, Korea). The cDNA (2 μL) for autophagy-related proteins were amplified using the primers described in Supplementary Table 2. Following the manufacturer’s inscructions, the mRNA expression levels of target genes were analyzed by real-time PCR using a Rotor-Gene 6000 real-time thermal cycling system (Corbett Research, New South Wales, Australia) with a QuantiMix SYBR Kit (PhileKorea Technology, Daejeon, Korea). β-Actin was used as an internal control.

### Small interfering RNA (siRNA) transfection

Cells were grown until 70% confluence and transfected for 36 h with ON-TARGETplus siRNAs (GE Dharmacon, Lafayette, CO, USA) mixed by siRNA specific for eIF2α or non-targeting (*nt*) siRNA as a negative control with TurboFect Transfection Reagents (Thermo Fisher Scientific, Waltham, MA, USA) according to the manufacturer’s instructions. The siRNA efficacy for eIF2α was determined by western blot (Supplementary Fig. 2).

### Bacterial strains, plasmids, and complementation of the vvhA mutant

The strains and plasmid used in the present study are listed in Supplementary Table 1. All *V. vulnificus* strains are isogenic and naturally resistant to polymyxin B. Bacterial strains were cultured in LB medium supplemented with 2.0% (w/v) NaCl at 30 °C until mid-log phase (A_600_ = 0.500) and then harvested. The pellet was washed with PBS and prepared to desired CFU/mL. To complement the *vvhA* mutation, an open reading frame (ORF) of *vvhA* was amplified from the genomic DNA of *V. vulnificus* MO6-24/O by PCR with the primer pair VVHA001F and VVHA001R and then digested with BamHI (Supplementary Table 3). The amplified *vvhA* ORF was subcloned into the broad-host-range vector pRK415 linearized with the same enzyme to result in pKK1449^55^. *E. coli* S17-1 λ pir, tra strain containing pKK1449 was used as a conjugal donor to *vvhA* mutant^56^. The plasmid pKK1449 was delivered into the *vvhA* mutant by conjugation as described previously^57^.

### Animal models

All animal experiments were conducted following the National Institute of Health Guidelines and approved by the Institutional Animal Care and Use Committee at Seoul National University (SNU-150818-2). Seven-week-old ICR mice (n = 7) received intragastric inoculation of boiled *V. vulnificus* (Cont), *V. vulnificus* (WT), *V. vulnificus (vvhA* mutant), a mutant strain deficient in the *vvhA* gene and *V. vulnificus (vvhA* comp), the *vvhA* mutant strain complemented with the functional *vvhA* gene, at 1.1 × 10^9^ CFU/mL. We euthanized the mice 18 h after the inoculation. To evaluate the intestinal paracellular permeability, we examined the translocation of FITC-labeled 4-kDa dextran into the blood stream. Mice were received oral administration of FITC-dextran in PBS (20 mg/100 μL) for 4 h before being sacrificed. A blood sample was collected from retro-orbital plexus under deep anesthesia, and the concentration of FITC-dextran in plasma was measured with a Victor3 luminometer (Perkin-Ehmer Inc., Waltham, MA, USA) using 488 nm excitation and 515 nm emission. The ileum tissue of mice was obtained for analysis. For histopathological findings, half ileum tissues were embedded in O.C.T. compound. The tissues were then cut into 10-μm-thick frozen sections and processed for H&E staining. The other half were washed with cold PBS and stored at −70 °C for protein analysis.

### Statistical analysis

Results are expressed as mean ± standard errors (S.E.). Statistical analyses were performed by the ANOVA, followed in some cases by a comparison of treatment means with a control using the Bonferroni t-test. Differences were considered statistically significant at P < 0.05.

## Additional Information

**How to cite this article**: Song, E. J. *et al. Vibrio vulnificus* VvhA induces autophagy-related cell death through the lipid raft-dependent c-Src/NOX signaling pathway. *Sci. Rep.* **6**, 27080; doi: 10.1038/srep27080 (2016).

## Supplementary Material

Supplementary Information
