# Supplementary material for: Vibrio vulnificus VvhA induces autophagy-related cell death through the lipid raft-dependent c-Src/NOX signaling pathway
Source: Sci Rep. 2016 Jun 2;6:27080. doi: 10.1038/srep27080 (PMC4890043; doi:10.1038/srep27080)
Supplement: Supplementary Information [file srep27080-s1.pdf]

***Vibrio vulnificus* VvhA induces autophagy-related cell death through the lipid raft-dependent c-Src/NOX signaling pathway**

Eun Ju Song[1, +], Sei-Jung Lee[1, +], Hyeon Su Lim[1], Jun Sung Kim[1], Kyung Ku Jang[2], Sang Ho Choi[2], and Ho Jae Han[1,]*

## Supplementary Materials and Methods

**Purification of the recombinant haemolysin VvhA.** To find the molecular role of VvhA in Caco-2 cells, we prepared a recombinant protein of VvhA (rVvhA) following the method of Lee et al.[1]. Briefly, the coding region of *vvhA* was amplified and then cloned into a His6-tag expression vector, pET29a(+) (Novagen, Madison, WI, USA), resulting in pKS1201 (Supplementary Table S1). *E. coli* BL21 (DE3) carrying the pKS1201 was grown in LB-ampicillin media at 37 °C until reaching an $A_{600}$ between 0.5 and 0.6. For inducing the protein expression, the temperature was lowered to 30 °C and the culture treated with 1 mM isopropyl-β-D-thiogalactopyranoside for 6 h. The cells were harvested and prepared for isolating the soluble fraction. Cell lysate containing $His_6$-tagged VvhBA protein was mixed with 1 ml of Ni-NTA agarose (Qiagen, Valencia, CA) for 1 h at 4 °C and the mixture was loaded on Bio-Spin Chromatography Columns (Bio-Rad Laboratories, Hercules, CA, USA). The resin was washed with buffer A, and bound VvhBA protein was eluted with buffer A containing 300 mM imidazole. After purification, the homogeneity of VvhBA was assessed by 12 % SDS-PAGE and Coomassie Blue staining. Purified proteins containing rVvhA was dialyzed against 20 mM Tris-Cl, pH 8.0, concentrated to 0.3 mg/mL using Slide-A-Lyzer Dialysis Cassettes (Thermo Scientific, Hudson, NH, USA), and stored at −80 °C until use.

**Bacterial strains, plasmids, and culture media.** The strains and plasmid used in the present study

are listed in Supplementary Table S1. All *V. vulnificus* strains (M06-24/O WT and M06-24/O *vvhA*) are isogenic and naturally resistant to polymyxin B. *V. vulnificus* strains were cultured in LB medium supplemented with 2.0 % (w/v) NaCl (LBS) at 30 °C. All media components were obtained from Difco Laboratories (Detroit, MI, USA). The bacterial strains were grown to mid-log phase ($A_{600}$ = 0.500) corresponding 2 × $10^8$ CFU/mL and centrifuged at 6000 × g for 5 min. The pellet was washed with PBS and prepared to desired colony-forming unit (CFU)/mL based on the $A_{600}$ determined using a UV–VIS spectrophotometer (UV-1800, Shimadzu, Japan).

**Complementation of the *vvhA* mutant**. To complement the *vvhA* mutation, an open reading frame (ORF) of *vvhA* was amplified from the genomic DNA of *Vibrio vulnificus* MO6-24/O by PCR with the primer pair VVHA001F and VVHA001R (Supplementary Table S2) and then digested with BamHI. The amplified *vvhA* ORF was subcloned into the broad-host-range vector pRK415 linearized with the same enzyme (Supplementary Table S1) to result in pKK1449[2]. *Escherichia coli* S17-1 *λ pir, tra* strain containing pKK1449 was used as a conjugal donor to *vvhA* mutant[3]. The plasmid pKK1449 was delivered into the *vvhA* mutant by conjugation as described previously[4].

## References for Supplementary


1. Lee, S. et al. *Vibrio vulnificus* VvhA induces NF-κB-dependent mitochondrial cell death via lipid raft-mediated ROS production in intestinal epithelial cells. Cell death & disease 6, 1655 (2015).

2. Keen, N., Tamaki, S., Kobayashi, D. & Trollinger, D. Improved broad-host-range plasmids for DNA cloning in gram-negative bacteria. Gene 70, 191-197 (1988).

3. Simon, R., Priefer, U. & Pühler, A. A broad host range mobilization system for invivo genetic-engineering-transposon mutagenesis in gram-negative bacteria. Bio-technology 1 (1983).

4. Park, J. H. et al. Correction: The cabABC Operon Essential for Biofilm and Rugose Colony Development in *Vibrio vulnificus*. PLoS pathogens 11, e1005252-e1005252 (2015).

**Supplementary Table 1. Plasmids and bacterial strains used in this study**

| Strain or plasmid | Relevant characteristics [a] | Reference or source |
|---|---|---|
| **Bacterial strains** | | |
| *V. vulnificus* | | |
| M06-24/O | Clinical isolate; virulent; WT | Laboratory collection |
| *E. coli* | | |
| BL21 (DE3) | $F^-$ *ompT hsdS$_B$* ($r_B^-m_B^-$) *gal dcm* (DE3) | Laboratory collection |
| S17-1λ*pir* | λ-*pir* lysogen; *thi pro hsdR hsdM*$^+$ *recA* RP4-2 Tc::Mu-Km::Tn7;$Tp^r$ $Sm^r$; host for π-requiring plasmids; conjugal donor | Simon *et al*., 1983[3] |
| **Plasmids** | | |
| pET29a(+) | $His_6$ tag fusion expression vector; $Km^r$ | Novagen |
| pKS1201 | pET29a(+) with VvhBA; $Km^r$ | Lee *et al*., 2015[1] |
| pRK415 | IncP *ori*; broad-host-range vector, *oriT* of RP4; $Tc^r$ | Keen *et al*., 1988[2] |
| pKK1449 | pRK415 with *vvhA*; $Tc^r$ | This study |

[a] $Tp^r$, trimethoprim resistant; $Sm^r$, streptomycin resistant; $Tc^r$, tetracycline resistant

**Supplementary Table 2. Sequences of primer pairs used in this study.**

| Genes | Forward primer/reverse primer |
|---|---|
| ULK1 | 5’-CAGAACTACCAGCGCATTGA-3’<br>5’-TCCACCCAGAGACATCTTCC-3’ |
| ULK2 | 5’-CTCCTCAGGTTCTCCAGTGC-3’<br>5’-TTGGTGGGAGAAGTTCCAAG-3’ |
| ATG14 | 5’-TCACCATCCAGGAACTCACA-3’<br>5’-TTCAGTCTTCGGCTGAGGTT-3’ |
| VPS34 | 5’-ATGTGTATGGTCCCGGAAAA-3’<br>5’-TGTCGATGAGCTTTGGTGAG-3’ |
| BECLIN-1 | 5’-GCCCTTTGAAGTACGAGCAG-3’<br>5’-GAGATCATCCCACCTGCACT-3’ |
| ATG5 | 5’-CGGGAACACCAAGTTTCACT-3’<br>5’-TCTGGGGAGACATCCGTAAG-3’ |
| ATG12 | 5’-TGGGATTGCAAAATGACAGA-3’<br>5’-TTCCCCATCTTCAGGATCAA-3’ |
| ATG16L1 | 5’-GTCTTCGATGCACATGATGG-3’<br>5’-GATTCGGCTTGCAAAATCAT-3’ |
| LC3B | 5’-AGCAGCATCCAACCAAAATC-3’<br>5’-CTGTGTCCGTTCACCAACAG-3’ |
| P62/SQSTM1 | 5’-CACCTGTCTGAGGGCTTCTC-3’<br>5’-CACACTCTCCCCAACGTTCT-3’ |
| RAB7 | 5’-CTGACCAAGGAGGTGATGGT-3’<br>5’-CTGGCCTGGATGAGAAACTC-3’ |
| FYCO1 | 5’-GGAGCTAGGAGCAGCAGAGA-3’<br>5’-CGCATCACTGGGAATAGGTT-3’ |
| LAMP1 | 5’-CTTCAGCAGGGGAGAGACAC-3’<br>5’-TGTTGGGGTTGATGTTGAGA-3’ |
| LAMP2 | 5’-GGTTAATGGCTCCGTTTTCA-3’<br>5’-ATGGGCACAAGGAAGTTGTC-3’ |
| β-Actin | 5’-AACCGCGAGAAGATGACCCAGATCATGTTT-3’<br>5’-AGCAGCCGTGGCCATCTCTTGCTCGAAGTC-3’ |

**Supplementary Table 3. Oligonucleotides used in this study**

| Name | Oligonucleotide Sequence (5' → 3')[a, b] | Use |
|---|---|---|
| **For mutant complementation** | | |
| VVHA001F | GGATCCAACCCTTACTCGTAATG | Amplification of the *vvhA* ORF |
| VVHA001R | GGATCCCTAGAGTTTGACTTGTTG | |

[a] The oligonucleotides were designed using the *V. vulnificus* MO6-24/O genomic sequence (GenBank™ accession number CP002469 and CP002470, www.ncbi.nlm.nih.gov).

[b] Regions of oligonucleotides not complementary to the corresponding genes are underlined.

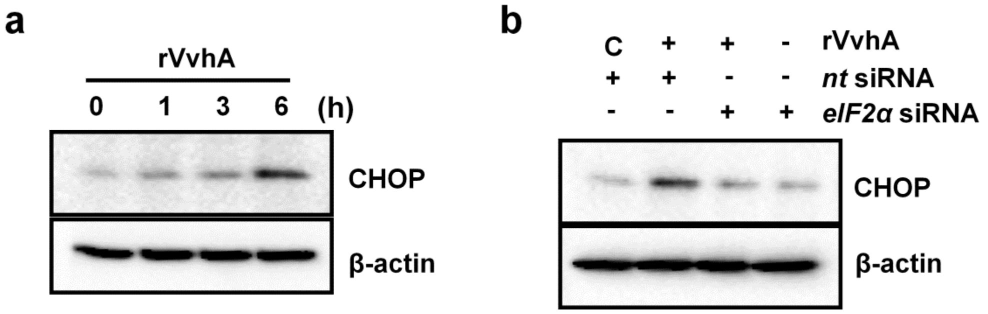


**Supplementary Figure 1. Effect of rVvhA on ER stress.**

**(a)** Time responses of rVvhA in the expression of CHOP were confirmed by western blotting. n = 3. **(b)** Cells were transfected for 36 h with *eIF2α* or *non-targeting* (*nt*) siRNA using TurboFect Transfection Reagent prior to rVvhA exposure for 6 h. CHOP expression was analyzed by using Western blot. n = 3.

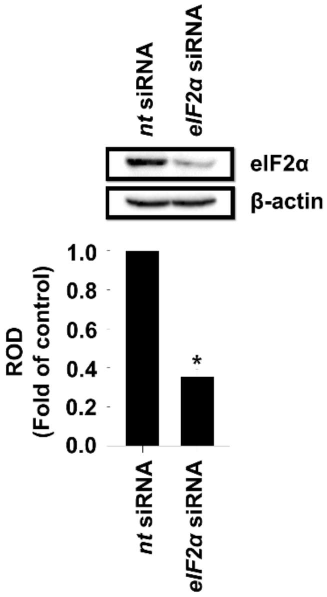


**Supplementary Figure 2. Effect of siRNA on target protein.** Cells were transfected for 36 h with *eIF2α* or *non-targeting* (*nt*) siRNA using TurboFect Transfection Reagent. Protein expressions were analyzed by using Western blot. The knockdown efficacies of eIF2α was 65%. Error bars represent the means ± S.E. *$P$ < 0.05 vs *nt* siRNA
